# Crystal structure of the Al_8_Cr_5_-type inter­metallic Al_7.85_Cr_5.16_

**DOI:** 10.1107/S2414314620004228

**Published:** 2020-04-09

**Authors:** Xu Geng, Bin Wen, Changzeng Fan

**Affiliations:** a State Key Laboratory of Metastable Materials Science and Technology, Yanshan University, Qinhuangdao 066004, People’s Republic of China; Benemérita Universidad Autónoma de Puebla, México

**Keywords:** crystal structure, high-pressure sinter­ing, inter­metallic, γ-brass phase

## Abstract

An aluminium-deficient Al_8_Cr_5_-type inter­metallic with formula Al_7.85_Cr_5.16_ (octa­aluminium penta­chromium) was uncovered when high-pressure sinter­ing of a mixture with composition Al_11_Cr_4_ was carried out. Structure analysis reveals that there are three co-occupied positions with refined occupancy factors for Al atoms being 0.958, 0.772 and 1/2. The present phase is confirmed to be isotypic with the previously reported rhombohedral Al_8_Cr_5_ ordered phase [Bradley & Lu (1937). *Z. Kristallogr.* **96**, 20–37] and structurally closely related to the disordered phases of rhombohedral Al_16_Cr_9.5_ and cubic Al_8_Cr_5_.

## Abstract 2

An aluminium-deficient γ-brass phase, Al_7.85_Cr_5.16_, was synthesized by high-pressure sinter­ing and its crystal structure determined.

## Graphical abstract

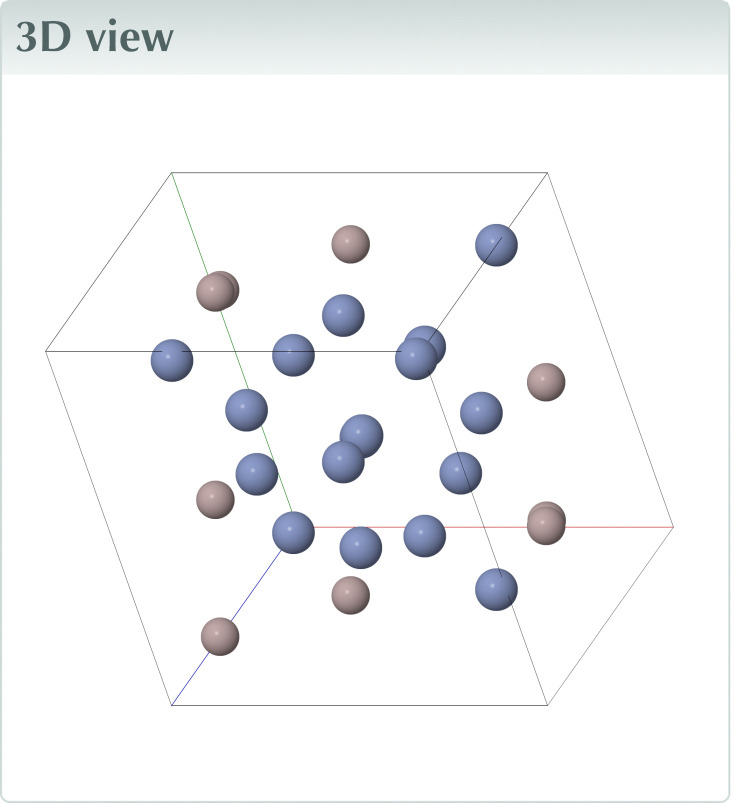

## Structure description

The γ_2_-Al_8_Cr_5_ phase (hereafter named as the γ_2_ phase) was determined to have a γ-brass-like structure by powder diffraction photographs. This phase was found in slowly cooled chromium–aluminium alloys (Bradley & Lu, 1937 ▸). Although the same clusters of 26 atoms are found in the γ_2_ phase, the atomic arrangement in the γ_2_ phase is much more complex than that of the γ-brass, and results in a rhombohedral rather than cubic symmetry (Bradley & Lu, 1937 ▸). A high-temperature γ_1_ phase was also reported to be stable between 1350 and 980°C at the same composition (Bradley & Lu, 1937 ▸) and its structure has been redetermined by single-crystal methods for a sample sintered at 1000°C for 6 h and re-annealed at 1215°C for 287 h (Brandon *et al.*, 1977 ▸). As a result of the close agreement of Brandon’s analysis with that of Bradley & Lu, it was suggested that either the structure of γ_1_ and γ_2_ are very similar, or that in the former case the crystals decomposed to γ_2_ on quenching. In another work, the high-temperature γ_1_ phase prepared by splat cooling was reported to be of the same type as Cu_5_Zn_8_, by using power diffraction data combined with electron diffraction patterns (Braun *et al.*, 1992 ▸). When comparing the three aforementioned models (see Table S1 of the supporting information), it was found that there are one vacancy position and three co-occupied positions in the Brandon model, while all atomic sites are fully occupied in Bradley & Lu’s model. For the convenience of comparison, the cubic Braun model was transformed to the rhombohedral description, and it was found that there are two co-occupied positions. In the study reported herein, the crystal structure of a third type of Al_8_Cr_5_ phase, with the refined chemical composition Al_7.85_Cr_5.16_ and hereafter named as γ_2_′-Al_8_Cr_5_ phase, was determined by single-crystal X-ray diffraction measurements.

**Figure 1:**
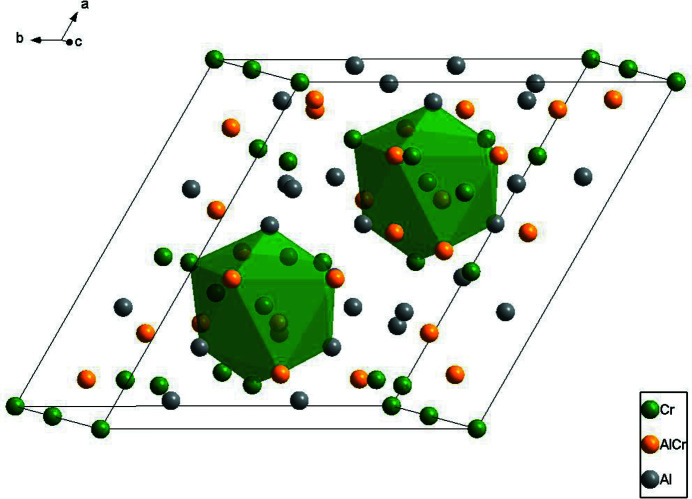
The crystal structure of Al_7.85_Cr_5.16_. The icosa­hedra centred on Cr4 are emphasized.

**Figure 2:**
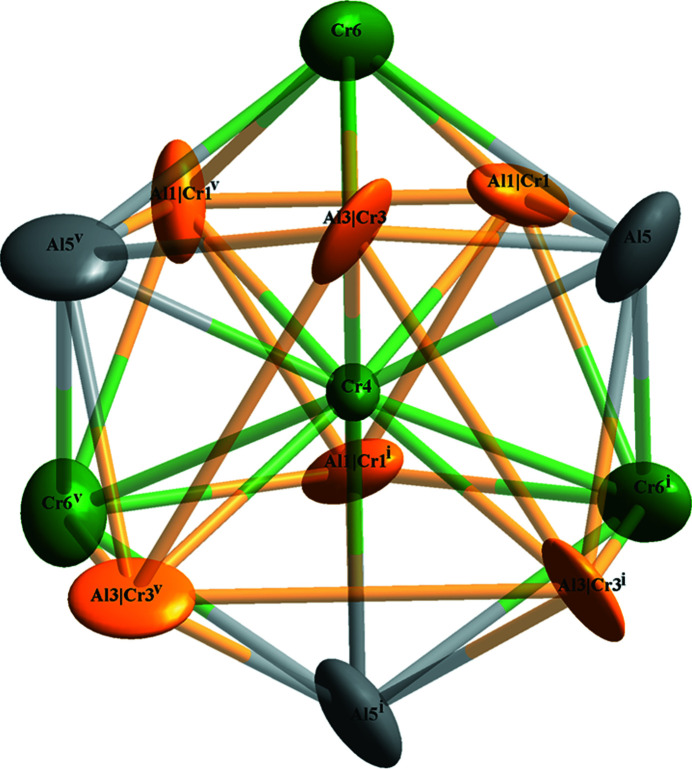
The environment of the Cr4 atom. Displacement ellipsoids are given at the 99% probability level. [Symmetry codes: (i) *y*, *z*, *x*; (v) *y*, *z*, *x*.]

Fig. 1 ▸ shows the crystal structure of γ_2_′-Al_8_Cr_5_ based on the standardized crystal data in the primitive trigonal setting (see Tables S2 and S3 of the supporting information). There are 78 atoms in the unit cell (*a* = *b* = 12.8717 Å, *c* = 7.8408 Å, *α* = *β* = 90°, *γ* = 120°), whose volume is three times that of the refined model (trigonal cell, rhombohedral axes, see Table 1 ▸). For simplicity, only two distorted icosa­hedra centred at Wyckoff sites 3*a* (Cr4, with coordinates 0, 0, *z*) are illustrated in Fig. 1 ▸, and the environment of the Cr4 atoms is shown in Fig. 2 ▸. The twelve vertices include three Al atoms (Al5), three Cr atoms (Cr6) along with six co-occupied Al/Cr sites (Al1/Cr1 and Al3/Cr3), for which the refined site occupancies converged to 0.772 (4) and 0.958 (4) for Al atoms Al1 and Al3.

**Figure 3:**
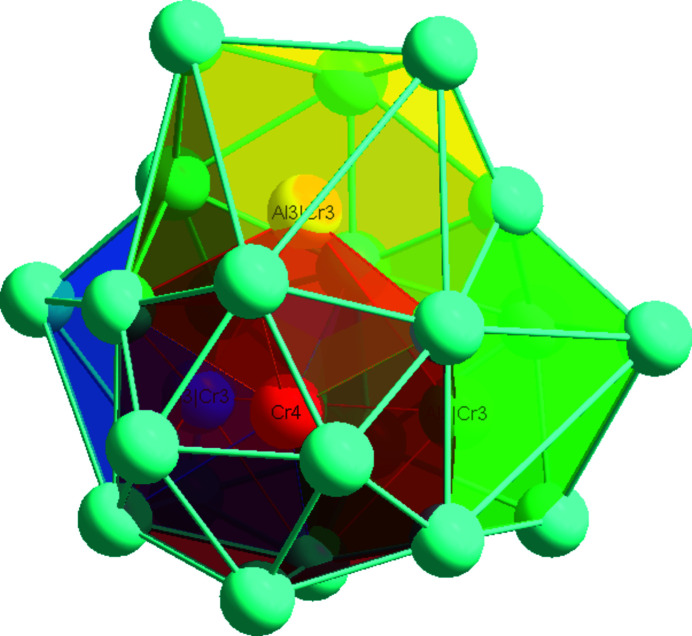
26-atom γ-brass-type cluster represented as four inter­penetrating distorted icosa­hedra centred at one Cr4 and three Al3/Cr3 sites.

**Figure 4:**
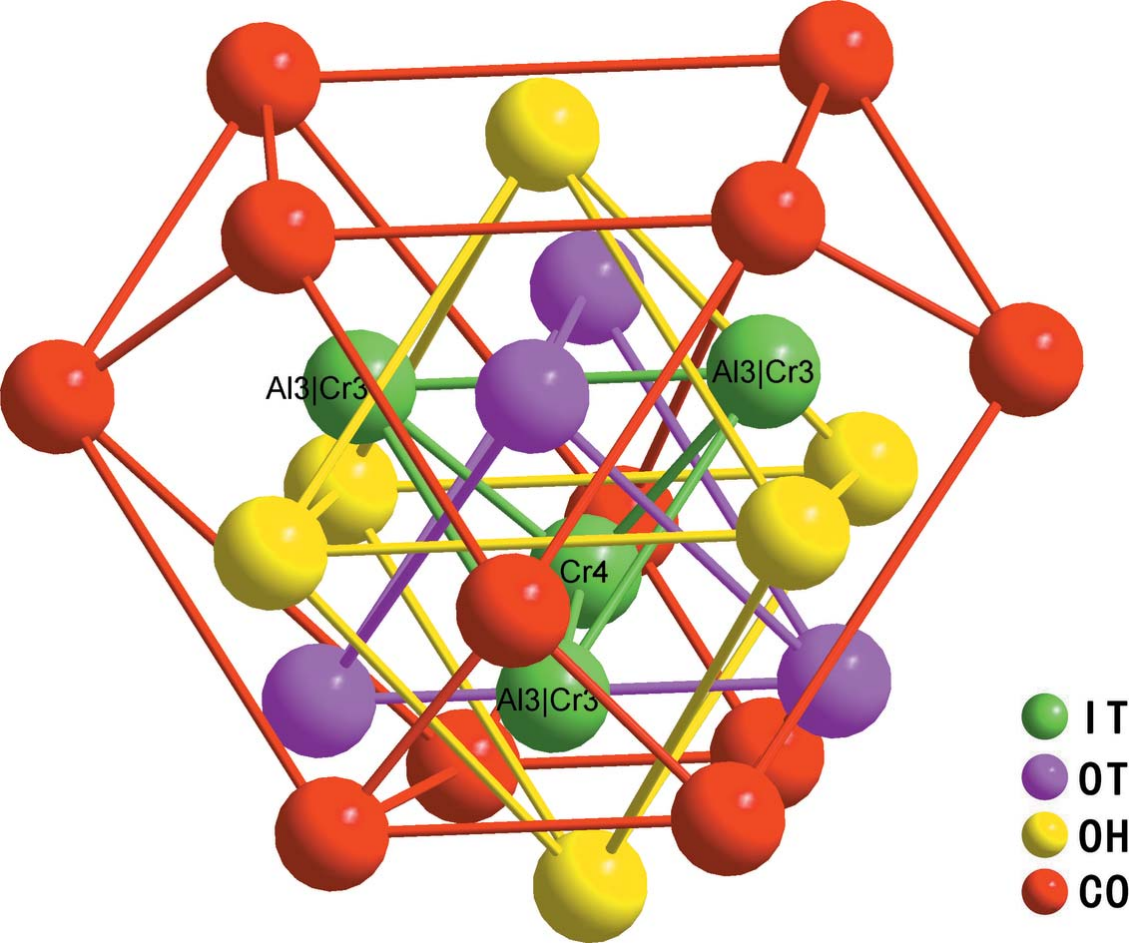
26-atom γ-brass-type cluster represented as a sequence of polyhedral shells.

The principle building blocks in the structure can also be represented by four inter­penetrating distorted icosa­hedra centred at one Cr4 and three Al3/Cr3 atomic sites, as shown in Fig. 3 ▸, similarly to the building blocks of the *I*-cell (space group *I* 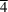 3*m*) of the γ-brass phase (Pankova *et al.*, 2013 ▸). According to the topological analysis of the structure model with the ‘nanocluster’ method available in the *ToposPro* package (Akhmetshina & Blatov, 2017 ▸), these one Cr4 and three Al3/Cr3 sites form an inner tetra­hedron (IT), followed by an outer tetra­hedron (OT), an octa­hedron (OH), whose vertices are projected onto the edges of the outer tetra­hedron, and finally a distorted cubocta­hedron (CO) with vertices located above the edges of the octa­hedron, as illustrated in Fig. 4 ▸.

The present rhombohedral γ_2_′-Al_8_Cr_5_ phase is thus confirmed to be isotypic to the previously reported ordered Al_8_Cr_5_ phase (Bradley & Lu, 1937 ▸), and closely related to the the disordered rhombohedral Al_16_Cr_9.5_ phase (Brandon *et al.*, 1977 ▸) and the disordered cubic Al_8_Cr_5_ phase (Braun *et al.*, 1992 ▸).

## Synthesis and crystallization

The high-purity elements Al (indicated purity 99.8%, 0.588 g) and Cr (indicated purity 99.95%, 0.539 g) were mixed uniformly in the stoichiometric ratio 11:4 and thoroughly ground in an agate mortar. The blended powders were then placed in a cemented carbide grinding mould of 5 mm diameter, and pressed into a tablet at about 4 MPa for 5 min. A cylindrical block (5 mm in diameter and 3 mm in height) was obtained without deformations or cracks. Details of the high-pressure sinter­ing experiment using a six-anvil high-temperature high-pressure apparatus can be found elsewhere (Liu & Fan, 2018 ▸). The samples were pressurized up to 5 GPa and heated to 1400°C for 30 minutes, slowly cooled to 660°C and held at this temperature for 2 h, and then rapidly cooled to room temperature by turning off the furnace power. Subsequently, a small amount of powder sample was uniformly placed on the inner wall of a quartz tube, annealed in a vacuum environment, heated to 300°C for 24 h, and then cooled within the furnace. A piece of a single crystal (0.13 × 0.06 × 0.05 mm^3^) was selected and mounted on a glass fibre for single-crystal X-ray diffraction measurements.

## Refinement

Table 1 ▸ shows the details of data collection and structural refinement. Three sites are co-occupied by Al and Cr atoms (Al1/Cr1, Al2/Cr2, Al3/Cr3). Site occupancies were refined, and then fixed to their as-found values, 0.772, 0.5 and 0.958 for Al1, Al2 and Al3, respectively, assuming full occupancy for each site. Atoms sharing the same site were constrained to have the same coordinates and displacement parameters. Moreover, disordered atoms were restrained to be isotropic, with standard deviations of 0.01 Å^2^ (Sheldrick, 2015*b* ▸). The maximum and minimum residual electron densities in the last difference map are located 1.68 Å from atom Cr3 and 0.36 Å from atom Cr4, respectively. The crystal was considered as a sample twinned by inversion (Parsons *et al.*, 2013 ▸), and the batch scale factor converged to *x* = 0.3 (2).

## Supplementary Material

Crystal structure: contains datablock(s) I. DOI: 10.1107/S2414314620004228/bh4049sup1.cif

Structure factors: contains datablock(s) I. DOI: 10.1107/S2414314620004228/bh4049Isup2.hkl

supporting information. DOI: 10.1107/S2414314620004228/bh4049sup3.pdf

CCDC reference: 1993113

Additional supporting information:  crystallographic information; 3D view; checkCIF report

## Figures and Tables

**Table 1:** Experimental details

Crystal data	
Chemical formula	Al_7.85_Cr_5.16_
*M*_r_	479.72
Crystal system, space group	Trigonal, *R*3*m*:*R*
Temperature (K)	296
*a* (Å)	7.8777 (5), 7.8777 (5)
α (°)	109.566 (2)
*V* (Å^3^)	375.01 (7)
*Z*	2
Radiation type	Mo *K*α
μ (mm^−1^)	8.05
Crystal size (mm)	0.13 × 0.06 × 0.05

Data collection	
Diffractometer	Bruker D8 Venture Photon 100 CMOS
Absorption correction	Multi-scan (*SADABS*; Bruker, 2015 ▸)
*T*_min_, *T*_max_	0.496, 0.523
No. of measured, independent and observed [*I* > 2σ(*I*)] reflections	7330, 576, 547
*R*_int_	0.090
(sin θ/λ)_max_ (Å^−1^)	0.634

Refinement	
*R*[*F* ^2^ > 2σ(*F* ^2^)], *wR*(*F* ^2^), *S*	0.068, 0.178, 1.16
No. of reflections	576
No. of parameters	53
No. of restraints	37
Δρ_max_, Δρ_min_ (e Å^−3^)	1.02, −1.27
Absolute structure	Refined as an inversion twin.
Absolute structure parameter	0.3 (2)

Computer programs: *APEX3* and *SAINT* (Bruker, 2015 ▸), *SHELXT2014/5* (Sheldrick, 2015*a* ▸), *SHELXL2017/1* (Sheldrick, 2015*b* ▸), *DIAMOND* (Brandenburg & Putz, 2017 ▸) and *publCIF* (Westrip, 2010 ▸).

## full crystallographic data

### Crystal data

**Table:**

Al_7.85_Cr_5.16_	*D*_x_ = 4.248 Mg m^−^^3^
*M**_r_* = 479.72	Mo *K*α radiation, λ = 0.71073 Å
Trigonal, *R*3*m*:*R*	Cell parameters from 2208 reflections
*a* = 7.8777 (5) Å	θ = 3.2–26.3°
α = 109.566 (2)°	µ = 8.05 mm^−^^1^
*V* = 375.01 (7) Å^3^	*T* = 296 K
*Z* = 2	Graininess, metallic silver
*F*(000) = 451	0.13 × 0.06 × 0.05 mm

### Data collection

**Table:**

Bruker D8 Venture Photon 100 CMOS diffractometer	547 reflections with *I* > 2σ(*I*)
φ and ω scans	*R*_int_ = 0.090
Absorption correction: multi-scan (SADABS; Bruker, 2015)	θ_max_ = 26.8°, θ_min_ = 3.2°
*T*_min_ = 0.496, *T*_max_ = 0.523	*h* = −9→9
7330 measured reflections	*k* = −9→9
576 independent reflections	*l* = −9→9

### Refinement

**Table:**

Refinement on *F*^2^	37 restraints
Least-squares matrix: full	*w* = 1/[σ^2^(*F*_o_^2^) + (0.0652*P*)^2^ + 19.1482*P*] where *P* = (*F*_o_^2^ + 2*F*_c_^2^)/3
*R*[*F*^2^ > 2σ(*F*^2^)] = 0.068	(Δ/σ)_max_ < 0.001
*wR*(*F*^2^) = 0.178	Δρ_max_ = 1.02 e Å^−^^3^
*S* = 1.16	Δρ_min_ = −1.27 e Å^−^^3^
576 reflections	Absolute structure: Refined as an inversion twin.
53 parameters	Absolute structure parameter: 0.3 (2)

### Special details

**Table:**

Refinement. Refined as a two-component inversion twin

### Fractional atomic coordinates and isotropic or equivalent isotropic displacement parameters (Å^2^)

**Table:**

	*x*	*y*	*z*	*U*_iso_*/*U*_eq_	Occ. (<1)
Al1	0.2998 (14)	0.2998 (14)	0.0928 (15)	0.0108 (19)	0.772
Cr1	0.2998 (14)	0.2998 (14)	0.0928 (15)	0.0108 (19)	0.228
Al2	1.3096 (13)	0.6646 (11)	0.6646 (11)	0.0117 (19)	0.5
Cr2	1.3096 (13)	0.6646 (11)	0.6646 (11)	0.0117 (19)	0.5
Al3	0.9238 (18)	0.6488 (14)	0.6488 (14)	0.012 (2)	0.958
Cr3	0.9238 (18)	0.6488 (14)	0.6488 (14)	0.012 (2)	0.042
Cr4	−0.0434 (12)	−0.0434 (12)	−0.0434 (12)	0.006 (2)	
Cr5	0.6391 (8)	0.3060 (8)	0.3060 (8)	0.0028 (10)	
Cr6	1.3001 (10)	0.9464 (8)	0.9464 (8)	0.0108 (14)	
Cr7	0.5198 (13)	0.5198 (13)	0.5198 (13)	0.008 (2)	
Al4	0.5500 (17)	−0.0607 (15)	0.2804 (16)	0.018 (2)	
Al5	0.0366 (18)	0.0366 (18)	−0.313 (2)	0.015 (3)	

### Atomic displacement parameters (Å^2^)

**Table:**

	*U*^11^	*U*^22^	*U*^33^	*U*^12^	*U*^13^	*U*^23^
Al1	0.007 (3)	0.007 (3)	0.019 (5)	0.005 (3)	0.006 (3)	0.006 (3)
Cr1	0.007 (3)	0.007 (3)	0.019 (5)	0.005 (3)	0.006 (3)	0.006 (3)
Al2	0.008 (4)	0.008 (3)	0.008 (3)	0.000 (3)	0.000 (3)	0.001 (3)
Cr2	0.008 (4)	0.008 (3)	0.008 (3)	0.000 (3)	0.000 (3)	0.001 (3)
Al3	0.013 (5)	0.010 (3)	0.010 (3)	0.007 (3)	0.007 (3)	−0.001 (3)
Cr3	0.013 (5)	0.010 (3)	0.010 (3)	0.007 (3)	0.007 (3)	−0.001 (3)
Cr4	0.007 (4)	0.007 (4)	0.007 (4)	0.005 (4)	0.005 (4)	0.005 (4)
Cr5	0.001 (2)	0.0034 (18)	0.0034 (18)	0.0001 (17)	0.0001 (17)	0.002 (2)
Cr6	0.016 (3)	0.008 (3)	0.008 (3)	0.006 (3)	0.006 (3)	0.003 (3)
Cr7	0.006 (3)	0.006 (3)	0.006 (3)	0.000 (4)	0.000 (4)	0.000 (4)
Al4	0.024 (5)	0.021 (4)	0.031 (5)	0.016 (3)	0.021 (4)	0.021 (4)
Al5	0.015 (4)	0.015 (4)	0.017 (5)	0.004 (5)	0.011 (4)	0.011 (4)

### Geometric parameters (Å, º)

**Table:**

Al1—Cr5	2.611 (7)	Cr2—Al5^xi^	2.930 (16)
Al1—Cr5^i^	2.611 (7)	Al3—Cr5	2.572 (12)
Al1—Cr6^ii^	2.634 (7)	Al3—Cr6	2.606 (11)
Al1—Cr6^iii^	2.634 (7)	Al3—Cr4^xiii^	2.655 (12)
Al1—Cr4	2.644 (11)	Al3—Cr7	2.678 (13)
Al1—Al4^i^	2.654 (11)	Al3—Al5^xiii^	2.758 (11)
Al1—Al4^iv^	2.654 (11)	Al3—Al5^xiv^	2.758 (11)
Al1—Al5	2.659 (17)	Al3—Al4^xii^	2.798 (12)
Al1—Al1^i^	2.665 (16)	Al3—Al4^ix^	2.798 (12)
Al1—Al1^v^	2.665 (16)	Cr3—Cr5	2.572 (12)
Al1—Cr7	2.739 (11)	Cr3—Cr6	2.606 (11)
Cr1—Cr5	2.611 (7)	Cr3—Cr4^xiii^	2.655 (12)
Cr1—Cr5^i^	2.611 (7)	Cr3—Cr7	2.678 (13)
Cr1—Cr6^ii^	2.634 (7)	Cr3—Al5^xiii^	2.758 (11)
Cr1—Cr6^iii^	2.634 (7)	Cr3—Al5^xiv^	2.758 (11)
Cr1—Cr4	2.644 (11)	Cr3—Al4^xii^	2.798 (12)
Cr1—Al4^i^	2.654 (11)	Cr3—Al4^ix^	2.798 (12)
Cr1—Al4^iv^	2.654 (11)	Cr4—Al5	2.616 (10)
Cr1—Al5	2.659 (17)	Cr4—Al5^v^	2.616 (10)
Cr1—Cr7	2.739 (11)	Cr4—Al5^i^	2.616 (10)
Al2—Cr6	2.604 (9)	Cr4—Cr6^iii^	2.761 (7)
Al2—Cr7^vi^	2.647 (9)	Cr4—Cr6^ii^	2.761 (7)
Al2—Al4^vii^	2.656 (9)	Cr5—Cr7	2.603 (7)
Al2—Al4^viii^	2.656 (9)	Cr5—Al4^xii^	2.620 (10)
Al2—Cr5^ix^	2.754 (6)	Cr5—Al4^ix^	2.620 (10)
Al2—Cr5^x^	2.754 (6)	Cr5—Al4^iv^	2.652 (9)
Al2—Al5^xi^	2.930 (16)	Cr5—Al4	2.652 (9)
Al2—Al4^xii^	2.996 (10)	Cr5—Al5^x^	2.683 (12)
Al2—Al4^ix^	2.996 (10)	Cr6—Al5^xi^	2.654 (12)
Cr2—Cr6	2.604 (9)	Cr6—Al4^xv^	2.739 (11)
Cr2—Cr7^vi^	2.647 (9)	Cr6—Al4^xiii^	2.739 (11)
Cr2—Al4^vii^	2.656 (9)	Cr6—Al5^xiv^	2.849 (8)
Cr2—Al4^viii^	2.656 (9)	Al4—Al5^x^	2.693 (12)
Cr2—Cr5^ix^	2.754 (6)	Al4—Al4^ix^	2.726 (5)
Cr2—Cr5^x^	2.754 (6)	Al4—Al4^xvi^	2.726 (5)
			
Cr5—Al1—Cr5^i^	110.4 (5)	Cr3—Cr5—Cr7	62.3 (4)
Cr5—Al1—Cr6^ii^	63.75 (14)	Al3—Cr5—Cr7	62.3 (4)
Cr5^i^—Al1—Cr6^ii^	167.7 (4)	Cr3—Cr5—Al1^v^	116.2 (4)
Cr5—Al1—Cr6^iii^	167.7 (4)	Al3—Cr5—Al1^v^	116.2 (4)
Cr5^i^—Al1—Cr6^iii^	63.75 (14)	Cr7—Cr5—Al1^v^	63.4 (3)
Cr6^ii^—Al1—Cr6^iii^	119.6 (5)	Cr7—Cr5—Al1	63.4 (3)
Cr5—Al1—Cr4	112.8 (3)	Al1^v^—Cr5—Al1	61.4 (4)
Cr5^i^—Al1—Cr4	112.8 (3)	Cr7—Cr5—Cr1	63.4 (3)
Cr6^ii^—Al1—Cr4	63.1 (2)	Cr3—Cr5—Al4^xii^	65.2 (3)
Cr6^iii^—Al1—Cr4	63.1 (2)	Al3—Cr5—Al4^xii^	65.2 (3)
Cr5—Al1—Al4^i^	125.5 (4)	Cr7—Cr5—Al4^xii^	110.8 (3)
Cr5^i^—Al1—Al4^i^	60.5 (2)	Al1^v^—Cr5—Al4^xii^	168.9 (3)
Cr6^ii^—Al1—Al4^i^	131.8 (4)	Al1—Cr5—Al4^xii^	107.8 (3)
Cr6^iii^—Al1—Al4^i^	62.4 (2)	Cr1—Cr5—Al4^xii^	107.8 (3)
Cr4—Al1—Al4^i^	120.0 (3)	Cr3—Cr5—Al4^ix^	65.2 (3)
Cr5—Al1—Al4^iv^	60.5 (2)	Al3—Cr5—Al4^ix^	65.2 (3)
Cr5^i^—Al1—Al4^iv^	125.5 (4)	Cr7—Cr5—Al4^ix^	110.8 (3)
Cr6^ii^—Al1—Al4^iv^	62.4 (2)	Al1^v^—Cr5—Al4^ix^	107.8 (3)
Cr6^iii^—Al1—Al4^iv^	131.8 (4)	Al1—Cr5—Al4^ix^	168.9 (3)
Cr4—Al1—Al4^iv^	120.0 (3)	Cr1—Cr5—Al4^ix^	168.9 (3)
Al4^i^—Al1—Al4^iv^	81.7 (5)	Al4^xii^—Cr5—Al4^ix^	83.0 (5)
Cr5—Al1—Al5	124.0 (2)	Cr3—Cr5—Al4^iv^	121.2 (3)
Cr5^i^—Al1—Al5	124.0 (2)	Al3—Cr5—Al4^iv^	121.2 (3)
Cr6^ii^—Al1—Al5	65.1 (3)	Cr7—Cr5—Al4^iv^	115.6 (3)
Cr6^iii^—Al1—Al5	65.1 (3)	Al1^v^—Cr5—Al4^iv^	110.7 (4)
Cr4—Al1—Al5	59.1 (3)	Al1—Cr5—Al4^iv^	60.5 (3)
Al4^i^—Al1—Al5	76.7 (3)	Cr1—Cr5—Al4^iv^	60.5 (3)
Al4^iv^—Al1—Al5	76.7 (3)	Al4^xii^—Cr5—Al4^iv^	62.3 (2)
Cr5—Al1—Al1^i^	108.1 (2)	Al4^ix^—Cr5—Al4^iv^	129.1 (2)
Cr5^i^—Al1—Al1^i^	59.3 (2)	Cr3—Cr5—Al4	121.2 (3)
Cr6^ii^—Al1—Al1^i^	111.0 (2)	Al3—Cr5—Al4	121.2 (3)
Cr6^iii^—Al1—Al1^i^	59.6 (2)	Cr7—Cr5—Al4	115.6 (3)
Cr4—Al1—Al1^i^	59.73 (18)	Al1^v^—Cr5—Al4	60.5 (3)
Al4^i^—Al1—Al1^i^	109.0 (2)	Al1—Cr5—Al4	110.7 (4)
Al4^iv^—Al1—Al1^i^	168.2 (3)	Cr1—Cr5—Al4	110.7 (4)
Al5—Al1—Al1^i^	110.2 (3)	Al4^xii^—Cr5—Al4	129.1 (2)
Cr5—Al1—Al1^v^	59.3 (2)	Al4^ix^—Cr5—Al4	62.3 (2)
Cr5^i^—Al1—Al1^v^	108.1 (2)	Al4^iv^—Cr5—Al4	111.7 (5)
Cr6^ii^—Al1—Al1^v^	59.6 (2)	Cr3—Cr5—Al5^x^	128.3 (4)
Cr6^iii^—Al1—Al1^v^	111.0 (2)	Al3—Cr5—Al5^x^	128.3 (4)
Cr4—Al1—Al1^v^	59.73 (18)	Cr7—Cr5—Al5^x^	169.4 (4)
Al4^i^—Al1—Al1^v^	168.2 (3)	Al1^v^—Cr5—Al5^x^	107.8 (3)
Al4^iv^—Al1—Al1^v^	109.0 (2)	Al1—Cr5—Al5^x^	107.8 (3)
Al5—Al1—Al1^v^	110.2 (3)	Cr1—Cr5—Al5^x^	107.8 (3)
Al1^i^—Al1—Al1^v^	60.000 (1)	Al4^xii^—Cr5—Al5^x^	76.8 (3)
Cr5—Al1—Cr7	58.2 (2)	Al4^ix^—Cr5—Al5^x^	76.8 (3)
Cr5^i^—Al1—Cr7	58.2 (2)	Al4^iv^—Cr5—Al5^x^	60.6 (3)
Cr6^ii^—Al1—Cr7	111.2 (3)	Al4—Cr5—Al5^x^	60.6 (3)
Cr6^iii^—Al1—Cr7	111.2 (3)	Cr3—Cr5—Al2^xvi^	64.3 (2)
Cr4—Al1—Cr7	110.2 (4)	Al3—Cr5—Al2^xvi^	64.3 (2)
Al4^i^—Al1—Cr7	111.1 (3)	Cr7—Cr5—Al2^xvi^	59.1 (2)
Al4^iv^—Al1—Cr7	111.1 (3)	Al1^v^—Cr5—Al2^xvi^	60.5 (3)
Al5—Al1—Cr7	169.4 (5)	Al1—Cr5—Al2^xvi^	110.8 (3)
Al1^i^—Al1—Cr7	60.89 (18)	Cr1—Cr5—Al2^xvi^	110.8 (3)
Al1^v^—Al1—Cr7	60.89 (18)	Al4^xii^—Cr5—Al2^xvi^	125.9 (4)
Cr5—Cr1—Cr5^i^	110.4 (5)	Al4^ix^—Cr5—Al2^xvi^	59.2 (3)
Cr5—Cr1—Cr6^ii^	63.75 (14)	Al4^iv^—Cr5—Al2^xvi^	170.8 (4)
Cr5^i^—Cr1—Cr6^ii^	167.7 (4)	Al4—Cr5—Al2^xvi^	67.3 (2)
Cr5—Cr1—Cr6^iii^	167.7 (4)	Al5^x^—Cr5—Al2^xvi^	123.0 (2)
Cr5^i^—Cr1—Cr6^iii^	63.75 (14)	Cr2—Cr6—Al1^xiii^	149.6 (2)
Cr6^ii^—Cr1—Cr6^iii^	119.6 (5)	Al2—Cr6—Al1^xiii^	149.6 (2)
Cr5—Cr1—Cr4	112.8 (3)	Cr3—Cr6—Al1^xiii^	108.9 (3)
Cr5^i^—Cr1—Cr4	112.8 (3)	Al3—Cr6—Al1^xiii^	108.9 (3)
Cr6^ii^—Cr1—Cr4	63.1 (2)	Al1^xiii^—Cr6—Al1^xiv^	60.8 (4)
Cr6^iii^—Cr1—Cr4	63.1 (2)	Cr2—Cr6—Al5^xi^	67.7 (4)
Cr5—Cr1—Al4^i^	125.5 (4)	Al2—Cr6—Al5^xi^	67.7 (4)
Cr5^i^—Cr1—Al4^i^	60.5 (2)	Cr3—Cr6—Al5^xi^	136.9 (4)
Cr6^ii^—Cr1—Al4^i^	131.8 (4)	Al3—Cr6—Al5^xi^	136.9 (4)
Cr6^iii^—Cr1—Al4^i^	62.4 (2)	Al1^xiii^—Cr6—Al5^xi^	108.0 (4)
Cr4—Cr1—Al4^i^	120.0 (3)	Al1^xiv^—Cr6—Al5^xi^	108.0 (4)
Cr5—Cr1—Al4^iv^	60.5 (2)	Al1^xiii^—Cr6—Al4^xv^	59.2 (3)
Cr5^i^—Cr1—Al4^iv^	125.5 (4)	Al1^xiv^—Cr6—Al4^xv^	107.4 (4)
Cr6^ii^—Cr1—Al4^iv^	62.4 (2)	Al5^xi^—Cr6—Al4^xv^	59.9 (3)
Cr6^iii^—Cr1—Al4^iv^	131.8 (4)	Cr2—Cr6—Al4^xiii^	96.4 (3)
Cr4—Cr1—Al4^iv^	120.0 (3)	Al2—Cr6—Al4^xiii^	96.4 (3)
Al4^i^—Cr1—Al4^iv^	81.7 (5)	Cr3—Cr6—Al4^xiii^	126.1 (2)
Cr5—Cr1—Al5	124.0 (2)	Al3—Cr6—Al4^xiii^	126.1 (2)
Cr5^i^—Cr1—Al5	124.0 (2)	Al1^xiii^—Cr6—Al4^xiii^	107.4 (4)
Cr6^ii^—Cr1—Al5	65.1 (3)	Al1^xiv^—Cr6—Al4^xiii^	59.2 (3)
Cr6^iii^—Cr1—Al5	65.1 (3)	Al5^xi^—Cr6—Al4^xiii^	59.9 (3)
Cr4—Cr1—Al5	59.1 (3)	Al4^xv^—Cr6—Al4^xiii^	106.5 (5)
Al4^i^—Cr1—Al5	76.7 (3)	Al1^xiii^—Cr6—Cr4^xiii^	58.6 (2)
Al4^iv^—Cr1—Al5	76.7 (3)	Al1^xiv^—Cr6—Cr4^xiii^	58.6 (2)
Cr5—Cr1—Cr7	58.2 (2)	Al5^xi^—Cr6—Cr4^xiii^	163.9 (4)
Cr5^i^—Cr1—Cr7	58.2 (2)	Al4^xv^—Cr6—Cr4^xiii^	113.1 (3)
Cr6^ii^—Cr1—Cr7	111.2 (3)	Al4^xiii^—Cr6—Cr4^xiii^	113.1 (3)
Cr6^iii^—Cr1—Cr7	111.2 (3)	Cr2—Cr6—Cr5^xiii^	127.0 (4)
Cr4—Cr1—Cr7	110.2 (4)	Al2—Cr6—Cr5^xiii^	127.0 (4)
Al4^i^—Cr1—Cr7	111.1 (3)	Cr3—Cr6—Cr5^xiii^	163.9 (3)
Al4^iv^—Cr1—Cr7	111.1 (3)	Al3—Cr6—Cr5^xiii^	163.9 (3)
Al5—Cr1—Cr7	169.4 (5)	Al1^xiii^—Cr6—Cr5^xiii^	57.7 (2)
Cr6—Al2—Cr7^vi^	150.7 (4)	Al1^xiv^—Cr6—Cr5^xiii^	57.7 (2)
Cr6—Al2—Al4^vii^	71.0 (2)	Al5^xi^—Cr6—Cr5^xiii^	59.2 (3)
Cr7^vi^—Al2—Al4^vii^	108.3 (2)	Al4^xv^—Cr6—Cr5^xiii^	57.6 (2)
Cr6—Al2—Al4^viii^	71.0 (2)	Al4^xiii^—Cr6—Cr5^xiii^	57.6 (3)
Cr7^vi^—Al2—Al4^viii^	108.3 (2)	Cr4^xiii^—Cr6—Cr5^xiii^	104.7 (3)
Al4^vii^—Al2—Al4^viii^	141.3 (4)	Cr2—Cr6—Al5^xiv^	99.6 (3)
Cr6—Al2—Cr5^ix^	128.85 (18)	Al2—Cr6—Al5^xiv^	99.6 (3)
Cr7^vi^—Al2—Cr5^ix^	57.6 (2)	Cr3—Cr6—Al5^xiv^	60.5 (3)
Al4^vii^—Al2—Cr5^ix^	159.6 (4)	Al3—Cr6—Al5^xiv^	60.5 (3)
Al4^viii^—Al2—Cr5^ix^	57.9 (2)	Al1^xiii^—Cr6—Al5^xiv^	105.5 (3)
Cr6—Al2—Cr5^x^	128.85 (18)	Al1^xiv^—Cr6—Al5^xiv^	57.8 (3)
Cr7^vi^—Al2—Cr5^x^	57.6 (2)	Al5^xi^—Cr6—Al5^xiv^	127.5 (2)
Al4^vii^—Al2—Cr5^x^	57.9 (2)	Al4^xv^—Cr6—Al5^xiv^	164.0 (4)
Al4^viii^—Al2—Cr5^x^	159.6 (4)	Al4^xiii^—Cr6—Al5^xiv^	72.2 (3)
Cr5^ix^—Al2—Cr5^x^	102.2 (4)	Cr4^xiii^—Cr6—Al5^xiv^	55.6 (2)
Cr6—Al2—Al5^xi^	56.9 (3)	Cr5^xiii^—Cr6—Al5^xiv^	111.8 (3)
Cr7^vi^—Al2—Al5^xi^	93.8 (4)	Cr5—Cr7—Cr5^v^	110.9 (2)
Al4^vii^—Al2—Al5^xi^	82.8 (3)	Cr5—Cr7—Cr5^i^	110.9 (2)
Al4^viii^—Al2—Al5^xi^	82.8 (3)	Cr5^v^—Cr7—Cr5^i^	110.9 (2)
Cr5^ix^—Al2—Al5^xi^	111.2 (3)	Cr5—Cr7—Al2^xviii^	166.5 (5)
Cr5^x^—Al2—Al5^xi^	111.2 (3)	Cr5^v^—Cr7—Al2^xviii^	63.27 (8)
Cr6—Al2—Al4^xii^	99.9 (3)	Cr5^i^—Cr7—Al2^xviii^	63.27 (8)
Cr7^vi^—Al2—Al4^xii^	103.8 (3)	Cr5—Cr7—Al2^xix^	63.27 (8)
Al4^vii^—Al2—Al4^xii^	57.30 (18)	Cr5^v^—Cr7—Al2^xix^	166.5 (5)
Al4^viii^—Al2—Al4^xii^	123.5 (4)	Cr5^i^—Cr7—Al2^xix^	63.27 (8)
Cr5^ix^—Al2—Al4^xii^	108.9 (3)	Cr5—Cr7—Al2^xvi^	63.27 (8)
Cr5^x^—Al2—Al4^xii^	54.7 (2)	Cr5^v^—Cr7—Al2^xvi^	63.27 (8)
Al5^xi^—Al2—Al4^xii^	139.6 (3)	Cr5^i^—Cr7—Al2^xvi^	166.5 (5)
Cr6—Al2—Al4^ix^	99.9 (3)	Al2^xviii^—Cr7—Al2^xvi^	119.39 (7)
Cr7^vi^—Al2—Al4^ix^	103.8 (3)	Al2^xix^—Cr7—Al2^xvi^	119.39 (7)
Al4^vii^—Al2—Al4^ix^	123.5 (4)	Cr5—Cr7—Al3	58.3 (2)
Al4^viii^—Al2—Al4^ix^	57.30 (18)	Cr5^v^—Cr7—Al3	124.25 (17)
Cr5^ix^—Al2—Al4^ix^	54.7 (2)	Cr5^i^—Cr7—Al3	124.25 (17)
Cr5^x^—Al2—Al4^ix^	108.9 (3)	Cr5—Cr7—Cr3	58.3 (2)
Al5^xi^—Al2—Al4^ix^	139.6 (3)	Cr5^v^—Cr7—Cr3	124.25 (17)
Al4^xii^—Al2—Al4^ix^	70.8 (4)	Cr5^i^—Cr7—Cr3	124.25 (17)
Cr6—Cr2—Cr7^vi^	150.7 (4)	Cr5—Cr7—Al3^i^	124.25 (17)
Cr6—Cr2—Al4^vii^	71.0 (2)	Cr5^v^—Cr7—Al3^i^	124.25 (17)
Cr7^vi^—Cr2—Al4^vii^	108.3 (2)	Cr5^i^—Cr7—Al3^i^	58.3 (2)
Cr6—Cr2—Al4^viii^	71.0 (2)	Al2^xviii^—Cr7—Al3^i^	64.46 (17)
Cr7^vi^—Cr2—Al4^viii^	108.3 (2)	Al2^xix^—Cr7—Al3^i^	64.46 (17)
Al4^vii^—Cr2—Al4^viii^	141.3 (4)	Al2^xvi^—Cr7—Al3^i^	135.3 (5)
Cr6—Cr2—Cr5^ix^	128.85 (18)	Al3—Cr7—Al3^i^	82.8 (4)
Cr7^vi^—Cr2—Cr5^ix^	57.6 (2)	Cr3—Cr7—Al3^i^	82.8 (4)
Al4^vii^—Cr2—Cr5^ix^	159.6 (4)	Cr5—Cr7—Al3^v^	124.25 (17)
Al4^viii^—Cr2—Cr5^ix^	57.9 (2)	Cr5^v^—Cr7—Al3^v^	58.3 (2)
Cr6—Cr2—Cr5^x^	128.85 (18)	Cr5^i^—Cr7—Al3^v^	124.25 (17)
Cr7^vi^—Cr2—Cr5^x^	57.6 (2)	Cr5—Cr7—Al1^v^	58.5 (2)
Al4^vii^—Cr2—Cr5^x^	57.9 (2)	Cr5^v^—Cr7—Al1^v^	58.5 (2)
Al4^viii^—Cr2—Cr5^x^	159.6 (4)	Cr5^i^—Cr7—Al1^v^	106.2 (4)
Cr5^ix^—Cr2—Cr5^x^	102.2 (4)	Al2^xviii^—Cr7—Al1^v^	110.2 (3)
Cr6—Cr2—Al5^xi^	56.9 (3)	Al2^xix^—Cr7—Al1^v^	110.2 (3)
Cr7^vi^—Cr2—Al5^xi^	93.8 (4)	Al2^xvi^—Cr7—Al1^v^	60.3 (3)
Al4^vii^—Cr2—Al5^xi^	82.8 (3)	Al3—Cr7—Al1^v^	108.7 (3)
Al4^viii^—Cr2—Al5^xi^	82.8 (3)	Cr3—Cr7—Al1^v^	108.7 (3)
Cr5^ix^—Cr2—Al5^xi^	111.2 (3)	Al3^i^—Cr7—Al1^v^	164.4 (4)
Cr5^x^—Cr2—Al5^xi^	111.2 (3)	Al3^v^—Cr7—Al1^v^	108.7 (3)
Cr5—Al3—Cr6	157.4 (4)	Cr5—Cr7—Al1^i^	106.2 (4)
Cr5—Al3—Cr4^xiii^	139.3 (5)	Cr5^v^—Cr7—Al1^i^	58.5 (2)
Cr6—Al3—Cr4^xiii^	63.3 (3)	Cr5^i^—Cr7—Al1^i^	58.5 (2)
Cr5—Al3—Cr7	59.4 (3)	Al2^xviii^—Cr7—Al1^i^	60.3 (3)
Cr6—Al3—Cr7	143.2 (4)	Al2^xix^—Cr7—Al1^i^	110.2 (3)
Cr4^xiii^—Al3—Cr7	79.9 (4)	Al2^xvi^—Cr7—Al1^i^	110.2 (3)
Cr5—Al3—Al5^xiii^	123.2 (3)	Al3—Cr7—Al1^i^	164.4 (4)
Cr6—Al3—Al5^xiii^	64.1 (3)	Cr3—Cr7—Al1^i^	164.4 (4)
Cr4^xiii^—Al3—Al5^xiii^	57.8 (3)	Al3^i^—Cr7—Al1^i^	108.7 (3)
Cr7—Al3—Al5^xiii^	97.1 (3)	Al3^v^—Cr7—Al1^i^	108.7 (3)
Cr5—Al3—Al5^xiv^	123.2 (3)	Al1^v^—Cr7—Al1^i^	58.2 (4)
Cr6—Al3—Al5^xiv^	64.1 (3)	Cr5^xvi^—Al4—Cr5	144.8 (4)
Cr4^xiii^—Al3—Al5^xiv^	57.8 (3)	Cr5^xvi^—Al4—Al1^v^	86.3 (4)
Cr7—Al3—Al5^xiv^	97.1 (3)	Cr5—Al4—Al1^v^	59.0 (3)
Al5^xiii^—Al3—Al5^xiv^	109.5 (6)	Cr5^xvi^—Al4—Al2^xx^	62.9 (2)
Cr5—Al3—Al4^xii^	58.2 (3)	Cr5—Al4—Al2^xx^	150.4 (5)
Cr6—Al3—Al4^xii^	105.2 (4)	Al1^v^—Al4—Al2^xx^	148.8 (4)
Cr4^xiii^—Al3—Al4^xii^	141.1 (3)	Cr5^xvi^—Al4—Al5^x^	145.4 (6)
Cr7—Al3—Al4^xii^	103.4 (4)	Cr5—Al4—Al5^x^	60.2 (3)
Al5^xiii^—Al3—Al4^xii^	83.5 (3)	Al1^v^—Al4—Al5^x^	106.3 (3)
Al5^xiv^—Al3—Al4^xii^	154.2 (5)	Al2^xx^—Al4—Al5^x^	102.4 (4)
Cr5—Al3—Al4^ix^	58.2 (3)	Cr5^xvi^—Al4—Al4^ix^	134.5 (5)
Cr6—Al3—Al4^ix^	105.2 (4)	Cr5—Al4—Al4^ix^	58.3 (4)
Cr4^xiii^—Al3—Al4^ix^	141.1 (3)	Al1^v^—Al4—Al4^ix^	103.5 (4)
Cr7—Al3—Al4^ix^	103.4 (4)	Al2^xx^—Al4—Al4^ix^	95.5 (4)
Al5^xiii^—Al3—Al4^ix^	154.2 (5)	Al5^x^—Al4—Al4^ix^	74.9 (5)
Al5^xiv^—Al3—Al4^ix^	83.5 (3)	Cr5^xvi^—Al4—Al4^xvi^	59.4 (3)
Al4^xii^—Al3—Al4^ix^	76.6 (5)	Cr5—Al4—Al4^xvi^	128.9 (4)
Cr5—Cr3—Cr6	157.4 (4)	Al1^v^—Al4—Al4^xvi^	102.1 (5)
Cr5—Cr3—Cr4^xiii^	139.3 (5)	Al2^xx^—Al4—Al4^xvi^	67.6 (4)
Cr6—Cr3—Cr4^xiii^	63.3 (3)	Al5^x^—Al4—Al4^xvi^	86.1 (5)
Cr5—Cr3—Cr7	59.4 (3)	Al4^ix^—Al4—Al4^xvi^	151.5 (6)
Cr6—Cr3—Cr7	143.2 (4)	Cr5^xvi^—Al4—Cr6^ii^	106.6 (5)
Cr4^xiii^—Cr3—Cr7	79.9 (4)	Cr5—Al4—Cr6^ii^	61.8 (2)
Cr5—Cr3—Al5^xiii^	123.2 (3)	Al1^v^—Al4—Cr6^ii^	58.4 (3)
Cr6—Cr3—Al5^xiii^	64.1 (3)	Al2^xx^—Al4—Cr6^ii^	132.5 (4)
Cr4^xiii^—Cr3—Al5^xiii^	57.8 (3)	Al5^x^—Al4—Cr6^ii^	58.5 (3)
Cr7—Cr3—Al5^xiii^	97.1 (3)	Al4^ix^—Al4—Cr6^ii^	116.5 (5)
Cr5—Cr3—Al5^xiv^	123.2 (3)	Al4^xvi^—Al4—Cr6^ii^	68.0 (3)
Cr6—Cr3—Al5^xiv^	64.1 (3)	Cr5^xvi^—Al4—Al3^xvi^	56.6 (3)
Cr4^xiii^—Cr3—Al5^xiv^	57.8 (3)	Cr5—Al4—Al3^xvi^	118.0 (5)
Cr7—Cr3—Al5^xiv^	97.1 (3)	Al1^v^—Al4—Al3^xvi^	97.2 (4)
Al5^xiii^—Cr3—Al5^xiv^	109.5 (6)	Al2^xx^—Al4—Al3^xvi^	62.7 (3)
Cr5—Cr3—Al4^xii^	58.2 (3)	Al5^x^—Al4—Al3^xvi^	147.5 (5)
Cr6—Cr3—Al4^xii^	105.2 (4)	Al4^ix^—Al4—Al3^xvi^	78.0 (4)
Cr4^xiii^—Cr3—Al4^xii^	141.1 (3)	Al4^xvi^—Al4—Al3^xvi^	110.9 (4)
Cr7—Cr3—Al4^xii^	103.4 (4)	Cr6^ii^—Al4—Al3^xvi^	153.0 (5)
Al5^xiii^—Cr3—Al4^xii^	83.5 (3)	Cr5^xvi^—Al4—Al2^xvi^	99.8 (4)
Al5^xiv^—Cr3—Al4^xii^	154.2 (5)	Cr5—Al4—Al2^xvi^	58.0 (2)
Cr5—Cr3—Al4^ix^	58.2 (3)	Al1^v^—Al4—Al2^xvi^	56.9 (3)
Cr6—Cr3—Al4^ix^	105.2 (4)	Al2^xx^—Al4—Al2^xvi^	120.5 (4)
Cr4^xiii^—Cr3—Al4^ix^	141.1 (3)	Al5^x^—Al4—Al2^xvi^	114.1 (4)
Cr7—Cr3—Al4^ix^	103.4 (4)	Al4^ix^—Al4—Al2^xvi^	55.1 (2)
Al5^xiii^—Cr3—Al4^ix^	154.2 (5)	Al4^xvi^—Al4—Al2^xvi^	153.3 (5)
Al5^xiv^—Cr3—Al4^ix^	83.5 (3)	Cr6^ii^—Al4—Al2^xvi^	106.8 (3)
Al4^xii^—Cr3—Al4^ix^	76.6 (5)	Al3^xvi^—Al4—Al2^xvi^	61.3 (4)
Al5—Cr4—Al5^v^	118.81 (14)	Cr4—Al5—Cr6^xxi^	152.4 (6)
Al5—Cr4—Al5^i^	118.81 (14)	Cr4—Al5—Cr1	60.2 (4)
Al5^v^—Cr4—Al5^i^	118.81 (14)	Cr6^xxi^—Al5—Cr1	147.5 (5)
Al5—Cr4—Cr1	60.7 (4)	Cr4—Al5—Al1	60.2 (4)
Al5^v^—Cr4—Cr1	112.2 (3)	Cr6^xxi^—Al5—Al1	147.5 (5)
Al5^i^—Cr4—Cr1	112.2 (3)	Cr4—Al5—Cr5^xix^	145.1 (6)
Al5—Cr4—Al1	60.7 (4)	Cr6^xxi^—Al5—Cr5^xix^	62.5 (3)
Al5^v^—Cr4—Al1	112.2 (3)	Cr1—Al5—Cr5^xix^	84.9 (5)
Al5^i^—Cr4—Al1	112.2 (3)	Al1—Al5—Cr5^xix^	84.9 (5)
Al5—Cr4—Al1^i^	112.2 (3)	Cr4—Al5—Al4^xxii^	125.1 (3)
Al5^v^—Cr4—Al1^i^	112.2 (3)	Cr6^xxi^—Al5—Al4^xxii^	61.6 (3)
Al5^i^—Cr4—Al1^i^	60.7 (4)	Al1—Al5—Al4^xxii^	102.9 (4)
Cr1—Cr4—Al1^i^	60.5 (4)	Cr5^xix^—Al5—Al4^xxii^	59.1 (3)
Al1—Cr4—Al1^i^	60.5 (4)	Cr4—Al5—Al4^xix^	125.1 (3)
Al5—Cr4—Al1^v^	112.2 (3)	Cr6^xxi^—Al5—Al4^xix^	61.6 (3)
Al5^v^—Cr4—Al1^v^	60.7 (4)	Cr1—Al5—Al4^xix^	102.9 (4)
Al5^i^—Cr4—Al1^v^	112.2 (3)	Al1—Al5—Al4^xix^	102.9 (4)
Cr1—Cr4—Al1^v^	60.5 (4)	Cr5^xix^—Al5—Al4^xix^	59.1 (3)
Al1—Cr4—Al1^v^	60.5 (4)	Al4^xxii^—Al5—Al4^xix^	109.2 (5)
Al1^i^—Cr4—Al1^v^	60.5 (4)	Cr4—Al5—Al3^iii^	59.1 (3)
Al5—Cr4—Al3^iii^	63.1 (2)	Cr6^xxi^—Al5—Al3^iii^	101.0 (4)
Al5^v^—Cr4—Al3^iii^	134.0 (6)	Cr1—Al5—Al3^iii^	103.8 (4)
Al5^i^—Cr4—Al3^iii^	63.1 (2)	Al1—Al5—Al3^iii^	103.8 (4)
Cr1—Cr4—Al3^iii^	107.2 (3)	Cr5^xix^—Al5—Al3^iii^	138.3 (3)
Al1—Cr4—Al3^iii^	107.2 (3)	Al4^xxii^—Al5—Al3^iii^	149.3 (6)
Al1^i^—Cr4—Al3^iii^	107.2 (3)	Al4^xix^—Al5—Al3^iii^	79.2 (3)
Al1^v^—Cr4—Al3^iii^	165.2 (4)	Cr4—Al5—Al3^ii^	59.1 (3)
Al5—Cr4—Al3^xvii^	134.0 (6)	Cr6^xxi^—Al5—Al3^ii^	101.0 (4)
Al5^v^—Cr4—Al3^xvii^	63.1 (2)	Cr1—Al5—Al3^ii^	103.8 (4)
Al5^i^—Cr4—Al3^xvii^	63.1 (2)	Al1—Al5—Al3^ii^	103.8 (4)
Cr1—Cr4—Al3^xvii^	165.2 (4)	Cr5^xix^—Al5—Al3^ii^	138.3 (3)
Al1—Cr4—Al3^xvii^	165.2 (4)	Al4^xxii^—Al5—Al3^ii^	79.2 (3)
Al1^i^—Cr4—Al3^xvii^	107.2 (3)	Al4^xix^—Al5—Al3^ii^	149.3 (6)
Al1^v^—Cr4—Al3^xvii^	107.2 (3)	Al3^iii^—Al5—Al3^ii^	79.9 (6)
Al3^iii^—Cr4—Al3^xvii^	83.6 (4)	Cr4—Al5—Cr6^ii^	60.5 (3)
Al5—Cr4—Al3^ii^	63.1 (2)	Cr6^xxi^—Al5—Cr6^ii^	126.6 (2)
Al5^v^—Cr4—Al3^ii^	63.1 (2)	Cr1—Al5—Cr6^ii^	57.0 (2)
Al5^i^—Cr4—Al3^ii^	134.0 (6)	Al1—Al5—Cr6^ii^	57.0 (2)
Cr1—Cr4—Al3^ii^	107.2 (3)	Cr5^xix^—Al5—Cr6^ii^	101.9 (3)
Al1—Cr4—Al3^ii^	107.2 (3)	Al4^xxii^—Al5—Cr6^ii^	66.8 (2)
Al1^i^—Cr4—Al3^ii^	165.2 (4)	Al4^xix^—Al5—Cr6^ii^	155.4 (6)
Al1^v^—Cr4—Al3^ii^	107.2 (3)	Al3^iii^—Al5—Cr6^ii^	117.2 (4)
Al3^iii^—Cr4—Al3^ii^	83.6 (4)	Al3^ii^—Al5—Cr6^ii^	55.4 (2)
Al3^xvii^—Cr4—Al3^ii^	83.6 (4)	Cr4—Al5—Cr6^iii^	60.5 (3)
Al5—Cr4—Cr6^iii^	63.93 (12)	Cr6^xxi^—Al5—Cr6^iii^	126.6 (2)
Al5^v^—Cr4—Cr6^iii^	168.5 (6)	Cr1—Al5—Cr6^iii^	57.0 (2)
Al5^i^—Cr4—Cr6^iii^	63.93 (12)	Al1—Al5—Cr6^iii^	57.0 (2)
Cr1—Cr4—Cr6^iii^	58.27 (18)	Cr5^xix^—Al5—Cr6^iii^	101.9 (3)
Al1—Cr4—Cr6^iii^	58.27 (18)	Al4^xxii^—Al5—Cr6^iii^	155.4 (6)
Al1^i^—Cr4—Cr6^iii^	58.27 (18)	Al4^xix^—Al5—Cr6^iii^	66.8 (2)
Al1^v^—Cr4—Cr6^iii^	107.8 (4)	Al3^iii^—Al5—Cr6^iii^	55.4 (2)
Al3^iii^—Cr4—Cr6^iii^	57.5 (3)	Al3^ii^—Al5—Cr6^iii^	117.2 (4)
Al3^xvii^—Cr4—Cr6^iii^	124.18 (16)	Cr6^ii^—Al5—Cr6^iii^	106.1 (4)
Al3^ii^—Cr4—Cr6^iii^	124.18 (16)	Cr4—Al5—Al2^xxi^	97.0 (4)
Al5—Cr4—Cr6^ii^	63.93 (12)	Cr6^xxi^—Al5—Al2^xxi^	55.3 (3)
Al5^v^—Cr4—Cr6^ii^	63.93 (12)	Cr1—Al5—Al2^xxi^	157.2 (5)
Al5^i^—Cr4—Cr6^ii^	168.5 (6)	Al1—Al5—Al2^xxi^	157.2 (5)
Cr1—Cr4—Cr6^ii^	58.27 (18)	Cr5^xix^—Al5—Al2^xxi^	117.9 (4)
Al1—Cr4—Cr6^ii^	58.27 (18)	Al4^xxii^—Al5—Al2^xxi^	90.1 (4)
Al1^i^—Cr4—Cr6^ii^	107.8 (4)	Al4^xix^—Al5—Al2^xxi^	90.1 (4)
Al1^v^—Cr4—Cr6^ii^	58.27 (18)	Al3^iii^—Al5—Al2^xxi^	59.8 (3)
Al3^iii^—Cr4—Cr6^ii^	124.18 (16)	Al3^ii^—Al5—Al2^xxi^	59.8 (3)
Al3^xvii^—Cr4—Cr6^ii^	124.18 (16)	Cr6^ii^—Al5—Al2^xxi^	113.8 (3)
Al3^ii^—Cr4—Cr6^ii^	57.5 (3)	Cr6^iii^—Al5—Al2^xxi^	113.8 (3)
Cr6^iii^—Cr4—Cr6^ii^	111.1 (2)		

Symmetry codes: (i) *z*, *x*, *y*; (ii) *x*−1, *y*−1, *z*−1; (iii) *z*−1, *x*−1, *y*−1; (iv) *x*, *z*, *y*; (v) *y*, *z*, *x*; (vi) *x*+1, *y*, *z*; (vii) *z*+1, *y*+1, *x*; (viii) *z*+1, *x*, *y*+1; (ix) *y*+1, *z*, *x*; (x) *z*+1, *x*, *y*; (xi) *z*+2, *x*+1, *y*+1; (xii) *y*+1, *x*, *z*; (xiii) *x*+1, *y*+1, *z*+1; (xiv) *y*+1, *z*+1, *x*+1; (xv) *x*+1, *z*+1, *y*+1; (xvi) *z*, *x*−1, *y*; (xvii) *y*−1, *z*−1, *x*−1; (xviii) *x*−1, *y*, *z*; (xix) *y*, *z*, *x*−1; (xx) *y*, *z*−1, *x*−1; (xxi) *y*−1, *z*−1, *x*−2; (xxii) *z*, *y*, *x*−1.
