# Supplementary material for: Crystal structure of the Al8Cr5-type inter­metallic Al7.85Cr5.16
Source: IUCrdata. 2020 Apr 9;5(Pt 4):x200422. doi: 10.1107/S2414314620004228 (PMC9462207; doi:10.1107/S2414314620004228)
Supplement: Supplementary file 3 [file x-05-x200422-sup3.pdf]

Crystal structure of the Al_8Cr_5-type intermetallic $Al_{7.85}Cr_{5.16}$

Xu Geng, Bin Wen and Changzeng Fan*

State Key Laboratory of Metastable Materials Science and Technology，Yanshan, University, Qinhuangdao 066004, People's Republic of China. Correspondence email: chzfan@ysu.edu.cn

Supplementary materials include:

Table S1 Crystallographic information of all reported Al_8Cr_5 phases

Table S2. Lattice parameters for the standardization of crystal data for the γ_2'-Al_8Cr_5 phase based on the refined crystal data

Table S3. The atomic positions for the standardization of crystal data for the γ_2'-Al_8Cr_5 phase based on the refined crystal data

Table S1 Crystallographic information of all reported Al_8Cr_5 phases

Phase	Space Group		Lattice Parameters		Wyckoff positions					Ref.
	Symbol	number	*a* (Å)	*α* (˚)	Atom site		*x*	*y*	*z*	
Al_8Cr_5	*I-43m*	217	9.0900	90.0000	Al1	24*g*	0.3087	0.3087	0.0394	[1]
(1992)					Al2	8*c*	0.1062	0.1062	0.1062	
					Cr1	8*c*	0.1062	0.1062	0.1062	
					Al3	12*e*	0.3522	0	0	
					Cr2	12*e*	0.3522	0	0	
					Cr3	8*c*	0.8300	0.8300	0.8300	
$Al_{16}Cr_{9.5}$	*R3m*	160	7.8110	109.1300	Al1	3*b*	-0.3608	-0.3608	-0.0095	[2]
(1977)					Al2	3*b*	0.0055	0.0055	-0.2096	
					Al3	3*b*	-0.2709	-0.2709	-0.6270	
					Al4	3*b*	0.3699	0.3699	0.0306	
					Al5	6*c*	-0.3179	0.2982	0.0533	
					Al6	3*b*	0.3608	0.3608	0.5720	
					Cr1	1*a*	0.1795	0.1795	0.1795	
					Cr2	3*b*	0.0055	0.0055	-0.2096	
					Cr3	1*a*	-0.3071	-0.3071	-0.3071	
					Cr4	3*b*	0.3699	0.3699	0.0306	
					Cr5	3*b*	0	0	0.3451	

Al_8Cr_5	*R3m*	160	7.8050	109.1270	Cr1	1*a*	0.1940	0.1940	0.1940	[3]
(1937)					Cr2	3*b*	0.0030	0.0030	0.7940	
					Cr3	3*b*	0.9980	0.9980	0.3400	
					Cr4	3*b*	0.3550	0.3550	0.0060	
					Al1	1*a*	0.6720	0.6720	0.6720	
					Al2	3*b*	0.6540	0.6540	0.0120	
					Al3	3*b*	0.3490	0.3490	0.5820	
					Al4	3*b*	0.7220	0.7220	0.3560	
					Al5	6*c*	0.0330	0.2880	0.6610	

Table S2. Lattice parameters for the standardization of crystal data for the γ_2'-Al_8Cr_5 phase based on the refined crystal data

**Chemical Formula**	**Al_8Cr_5**
Crystal system, space group	Hexagonal, *R3m*
a, *b*, *c*/Å	12.8717, 12.8717, 7.8408
α, *β*, *γ*/°	90, 90, 120
V/Å^3	1125.03

Table S3. The atomic positions for the standardization of crystal data for the γ_2'-Al_8Cr_5 phase based on the refined crystal data

**Label**	**Site**	*x*	*y*	*z*	U_{iso}*/U_{eq}	Occ. (<1)
**Al1**	9*b*	0.5977 (12)	0.4023 (12)	0.0591 (9)	0.010871	0.7720
**Cr1**	9*b*	0.5977 (12)	0.4023 (12)	0.0591 (9)	0.010871	0.2280
**Al2**	9*b*	0.2150 (11)	0.7850 (10)	0.0770 (7)	0.011734	0.5000
**Cr2**	9*b*	0.2150 (11)	0.7850 (10)	0.0770 (7)	0.011734	0.5000
**Al3**	9*b*	0.0917 (14)	0.9083 (13)	0.2161 (9)	0.012218	0.9580
**Cr3**	9*b*	0.0917 (14)	0.9083 (13)	0.2161 (9)	0.012218	0.0420
**Cr4**	3*a*	0.0000 (10)	0.0000 (10)	0.0000 (7)	0.005509	
**Cr5**	9*b*	0.4444 (7)	0.5556 (7)	0.2062 (5)	0.003182	
**Cr6**	9*b*	0.7846 (8)	0.2154 (7)	0.2256 (6)	0.011041	
**Cr7**	3*a*	0.0000 (11)	0.0000 (11)	0.4368 (8)	0.009053	
**Al4**	18*c*	0.3732 (14)	0.0161 (14)	0.0334 (10)	0.018686	
**Al5**	9*b*	0.5501 (15)	0.4499 (16)	0.3699 (11)	0.014880	

[1] Braun, J., Ellner, M. & Predel, B. (1992). *J. Alloys Compd*.**183**, 444–448.

[2] Brandon, J. K., Pearson, W. B., Riley, P. W., Chieh, C. & Stokhuyzen, R. (1977). *Acta Crystallogr. B*.**33**, 1088–1095.

[3] Bradley, A. J. & Lu, S. S. (1937). *Z. Kristallogr. Cryst. Mater*.**96**, 20–37.
